# Platinum nanoparticles induce damage to DNA and inhibit DNA replication

**DOI:** 10.1371/journal.pone.0180798

**Published:** 2017-07-12

**Authors:** Lukas Nejdl, Jiri Kudr, Amitava Moulick, Dagmar Hegerova, Branislav Ruttkay-Nedecky, Jaromir Gumulec, Kristyna Cihalova, Kristyna Smerkova, Simona Dostalova, Sona Krizkova, Marie Novotna, Pavel Kopel, Vojtech Adam

**Affiliations:** 1 Department of Chemistry and Biochemistry, Mendel University in Brno, Brno, Czech Republic; 2 Central European Institute of Technology, Brno University of Technology, Brno, Czech Republic; 3 Department of Pathological Physiology, Faculty of Medicine, Masaryk University, Brno, Czech Republic; Helsingin Yliopisto, FINLAND

## Abstract

Sparsely tested group of platinum nanoparticles (PtNPs) may have a comparable effect as complex platinum compounds. The aim of this study was to observe the effect of PtNPs in *in vitro* amplification of DNA fragment of phage λ, on the bacterial cultures (*Staphylococcus aureus*), human foreskin fibroblasts and erythrocytes. *In vitro* synthesized PtNPs were characterized by dynamic light scattering (PtNPs size range 4.8–11.7 nm), zeta potential measurements (-15 mV at pH 7.4), X-ray fluorescence, UV/vis spectrophotometry and atomic absorption spectrometry. The PtNPs inhibited the DNA replication and affected the secondary structure of DNA at higher concentrations, which was confirmed by polymerase chain reaction, DNA sequencing and DNA denaturation experiments. Further, cisplatin (CisPt), as traditional chemotherapy agent, was used in all parallel experiments. Moreover, the encapsulation of PtNPs in liposomes (LipoPtNPs) caused an approximately 2.4x higher of DNA damage in comparison with CisPt, LipoCisPt and PtNPs. The encapsulation of PtNPs in liposomes also increased their antibacterial, cytostatic and cytotoxic effect, which was determined by the method of growth curves on *S*. *aureus* and HFF cells. In addition, both the bare and encapsulated PtNPs caused lower oxidative stress (determined by GSH/GSSG ratio) in the human erythrocytes compared to the bare and encapsulated CisPt. CisPt was used in all parallel experiments as traditional chemotherapy agent.

## Introduction

The first anticancer drug cisplatin (*cis*-diamminedichloroplatinum(II)) was discovered in 1965 by Rosenberg during his studies on the effects of an electric current on bacterial growth [1]. The binding of cisplatin to DNA and the interactions with non-DNA targets with subsequent triggering of cell death through apoptosis, necrosis or both belong to the most important parameters of cisplatin cytotoxicity [2]. Since then, numerous platinum complexes of second generation (carboplatin, oxaliplatin, nedaplatin) and third generation (lobaplatin, heptaplatin) have been developed [3] and evaluated as anticancer agents [4, 5]. The undesired side effects can be eliminated by specific molecular interactions of a drug with cancer cells and by selective targeting, which can be done by their encapsulation in the molecules suitable for selective transportation. The most common transporters belong to functionalized polymers, nanoparticles with conjugated platinum drugs, carbon nanotubes and micelles. Among them, liposomes, which consist of a phospholipid bilayer surrounding an aqueous core, are a very important class of compounds [6]. Liposomes have interesting biological activities including effective drug loading capacity, biocompatibility and improved pharmacokinetics. The liposomal formulations of cisplatin and oxaliplatin (Lipoplatin^™^ and Lipoxal^™^) are now widely used to reduce drug toxicity and also improve drug targeting [7, 8]. Experimentally, many nanoparticles consisting of the polymers or copolymers and the Pt-based drugs have been tested. Among these, polyethylene glycol-functionalized poly-isobutylene-maleic acid copolymer can form complexes with cisplatin. The nanoparticles are internalized into the endolysosomal compartment of cancer cells releasing cisplatin in a pH-dependent manner. These nanoparticles have been developed for better antitumor efficacy, which was confirmed in a 4T1 breast cancer model *in vivo* with limited nephrotoxicity [9]. Furthermore, the nanoparticles treatment resulted in reduced systemic and nephrotoxicity, which was confirmed by the decreased biodistribution of platinum in kidney [10, 11]. The nanoparticles of oxaliplatin and carboplatin with polyisobutylene maleic acid copolymer with glucosamine and albumin-bound paclitaxel, respectively, were also tested [12, 13].

Plenty of studies discuss the effects of halogenated Pt salts and complexes and are reviewed elsewhere [14–16], whereas the effects of PtNPs are not mostly elucidated. It was found that PtNPs have anti-oxidant and anti-inflammatory effects. In this case, the platinum nanoparticles are able to scavenge superoxide anion radical (O_2_^-^) and hydroxyl radical (OH) [11, 17, 18] and this ability was also shown for the PtNPs within apoferritin [19, 20]. The ability of the PtNPs to scavenge the reactive oxygen species was studied in mouse ischemic brain. The treatment with PtNPs significantly improved motor function and greatly reduced the infarct volume, especially in the cerebral cortex [21, 22]. The effects of the PtNPs of sizes of 20, 100 and more than 100 nm on HT29 have been confirmed and have been found to cause DNA strand breaks in a concentration, time and size-dependent manner, but appear not to translocate into the nucleus or interact with mitochondria [23].

The interaction of Pt-attached iron oxide nanoparticles with DNA of was studied previously using gel electrophoresis and transmission electron microscopy. Two types of interactions were suggested in this case: the first interaction involved the direct linkage of DNA molecules onto the surface of the nanoparticles, while the second interaction was suggested to be the detachment of small PtNPs from the iron oxide support by the strong DNA interaction [24, 25]. On the other hand, the PtNPs did not show cytotoxicity in several different cultured cells (TIG-1, MI-38, MRC-5, HeLa, and HepG2) [17]. The contradiction of these studies indicates that the biological effects of PtNPs remains unclear and seems to be dependent on the particle size and most probably surface properties.

The interaction of different nanoparticles (NPs) with bacteria and fungi was studied by Chwalibog et al. and Sawosz et al. [26, 27]. It was found that silver, gold, and PtNPs, with negative zeta potential, had cell damaging properties on *Staphylococcus aureus* (Gram-positive bacteria) and *Candida albicans* (fungi). The analysis of morphological effects of the interaction of PtNPs with *Listeria monocytogenes* (Gram-positive) by transmission electron microscopy (TEM) revealed that the PtNPs entered into the cells of *Listeria monocytogenes*. The authors even stated that the nanoparticles were located close to DNA and probably bound to it. In the case of *Salmonella enteritidis* (Gram-negative), the PtNPs probably bound to DNA. This was studied by confocal microscopy with Nomarski contrast and fluorescent DAPI labelling. After washing and centrifugation, the PtNPs-DNA complexes were characterized. The results indicated that the bacteria could be used as a transporter to deliver PtNPs to the specific points in the body [27, 28].

In this work, we present our results on the preparation of liposome with encapsulated platinum derivatives both PtNPs and cisplatin (CisPt). The aim of this study was to investigate the effect of these PtNPs on *in vitro* amplification of DNA fragment of phage λ, and on the bacterial cultures (*Staphylococcus aureus*), human foreskin fibroblasts (HFF) and erythrocytes.

## Materials and methods

### Chemicals and material

Cholesterol, 1,2-dioleoyl-sn-glycero-3-phospho-rac-(1-glycerol) sodium salt, chloroform, cisplatin, PtCl_4_, polyvinylpyrrolidone (PVP, 40 k), HCl (37%, *w*/*w*), NaBH_4_ and water were purchased from Sigma-Aldrich (St. Louis, MO, USA) in ACS purity. Hydrogenated phosphatidylcholine from soybean was a gift from Lipoid GMBH (Ludwigshafen, Germany). The deionised water was prepared using Aqual 25 (Aqual, Brno, Czech Republic) and was further purified using apparatus MilliQ Direct QUV equipped with the UV lamp (Merck Millipore, Merck KgaA, Darmstadt, Germany). The pH of the solutions was measured using a pH meter WTW inoLab (WTW GmbH, Weilheim, Germany).

### Preparation of PtNPs

The PtNPs were prepared according to Oh et al. [29] with a slight modification. The PtNPs were also covered by polyvinylpyrrolidone (PVP), in order to prevent their aggregation [30]. Briefly, PtCl_4_ (0.034 g) was dissolved in acidic water (5 mL) with 16 μL of 37% HCl. The solution of PtCl_4_ (5 mL) was added, with stirring, to another solution of 0.135 g PVP in water (45 mL). The mixture was stirred for 1 h at 25°C. After the addition of NaBH_4_ (50 mg) the final colour of the solution became black. The mixture was stirred overnight. Prepared particles were characterized as described in S1 Text.

### Preparation of liposome film

The liposomes were prepared according to the published method [31] with necessary modifications [32]. Briefly, cholesterol (100 mg), 1,2-dioleoyl-sn-glycero-3-phospho-rac-(1-glycerol) sodium salt (100 mg) and phosphatidylcholine (100 mg) were dissolved in chloroform (4.5 mL). A lipid film was obtained by rotary evaporation of chloroform. The residual chloroform was removed by nitrogen.

### Preparation of liposome filled with PtNPs (LipoPtNPs)

The solutions containing 0, 0.015, 0.03131, 0.0625, 0.125, 0.25, 0.5 or 1 mL of platinum nanoparticles (PtNPs, 400 μg/mL of Pt) were diluted with water to 1 mL and added to liposome (20 mg). The samples were homogenized for 15 min in an ultrasonic bath Sonorex Digital 10P (Bandelin, Berlin, Germany). The homogenized mixtures were then heated and shaken for 15 min at 60°C using a Thermomixer Comfort (Eppendorf, Germany). The samples were then washed several times with water employing Amicon 3k centrifugation filters (Merck Millipore, Merck KgaA, Darmstadt, Germany) with a final sample volume of 1 mL [32].

### Preparation of liposome filled with cisplatin (LipoCisPt)

The solutions containing 0, 0.015, 0.03131, 0.0625, 0.125, 0.25, 0.5 or 1 mL of cisplatin (CisPt, 0.4 mg Pt per mL) were diluted with water to 1 mL and added to liposome (20 mg). The samples were prepared according to the same procedure described in paragraph above.

### Polymerase chain reaction (PCR)

*Taq* PCR kit was purchased from New England Biolabs (Ipswitch, MA, USA). The forward and reverse primers for the amplification of λ *xis* gene fragment were synthesized by Sigma-Aldrich and their sequences were 5'-CCTGCTCTGCCGCTTCACGC-3' and 5'-TCCGGATAAAAACGTCGATGACATTTGC-3', respectively. The volume of the reaction mixture was 25 μL, which was composed of 2.5 μL of 10× standard *Taq* reaction buffer, 0.5 μL of 1 mM deoxynucleotide solution, 0.5 μL of each of the primers (10 μM), 0.125 μL of *Taq* DNA polymerase; selected volume of water or drugs diluted with water (sterile, ACS purity, Sigma-Aldrich) and 0.5 μL of bacteriophage λ DNA.

The PCR was performed using Mastercycler ep realplex^4^ S (Eppendorf, AG, Hamburg, Germany) with the following cycling conditions: initial denaturation for 120 s at 95°C; 30 cycles of PCR (15 s at 95°C, 15 s at 64°C and 45 s at 72°C) with a final elongation for 5 min at 72°C. The obtained DNA fragments (498 bp) were purified by MinElute PCR Purification Kit (Qiagen, Germany). The DNA concentration was determined spectrophotometrically.

### Agarose gel electrophoresis

The amplified product was analysed using agarose gel electrophoresis and the working conditions were as follows: 2% agarose gel (High melt/Medium fragment, Mercury, San Diego, CA, USA) with 1× TAE buffer, 60 V and 160 min (Bio-Rad, Hercules, CA, USA). The 100 bp DNA ladder (New England Biolabs, Ispwich, MA, USA) was used as a molecule size marker. The bands were visualized using UV transilluminator at 312 nm (Vilber-Lourmat, Marne-la-Vallée Cedex, France).

The fluorescence intensity of the ethidium bromide was detected by In-Vivo Xtreme (Carestream Health Inc., Rochester, NY, USA). The parameters were as follows: Excitation filter: 520 nm; Emission filter: 600 nm; Exposure time: 6 s; Binning: 2 × 2, f-Stop: 1.1; and Field of View: 19.5 × 19.5 cm. The images were processed by Carestream Molecular Software. The fluorescence was quantified using software and the background was subtracted. The intensity of the fluorescence of ethidium bromide was directly proportional to the concentration of the DNA.

### DNA sequencing

The purified DNA fragments (50 μg/mL) were mixed with different concentrations of PtNPs (25, 50, 100, 200 and 400 μg/mLPt) in a ratio of 1:1 (*v*/*v*). The solutions of DNA with PtNPs were incubated for 1 h at 25°C (Thermomixer 5355, Eppendorf, Germany). To remove the excess of PtNPs, a dialysis was performed for 12 h at 6°C using 0.025 μm membrane filter (Millipore, Ireland) with ACS water.

### CisPt interactions with DNA

The solution of the DNA fragments (50 μg/mL) was mixed with different concentrations of cisplatin (25, 50, 100, 200 and 400 μg/mLPt) in a rate of 1:1 (v/v) in the environment of 10 mM NaClO_4_. The solutions of DNA with the drugs were incubated for 24 h at 37°C. To remove the excess of the platinum-based drug a dialysis was performed for 24 h at 6°C using a 0.025 μm membrane filter (Millipore, Ireland).

### Sequencing of platinated DNA

The conditions of sequencing reaction were as described elsewhere [33].

### Comet assay

The comet assay was performed according to Singh et al. [34] with small modifications [24, 35]. Conventional slides were immersed in 0.5% normal-melting agarose (*v*/*v*, CLP, San Diego, USA) and dried at 50°C. The cells and slides were handled under dimmed light conditions during the whole process. Briefly, 75 μL of 0.5% low-melting agarose was mixed with approximately 10000 cells suspended in 10 μL of PBS and layered onto the slides and immediately covered with coverslips. After agarose solidification (5 min at 4°C), the coverslips were removed and the slides were immersed overnight in dark at 4°C in freshly prepared lysing solution (2.5 M NaCl, 100 mM Na_2_EDTA, 10 mM Tris, pH 10) containing 1% Triton X-100 and 10% DMSO (*v*/*v*). After the lysis, the slides were placed on a horizontal gel electrophoresis unit filled with electrophoretic buffer (300 mM NaOH, 1mM Na_2_EDTA, pH > 13) and left there for 30 min in dark at 4°C for DNA unwinding. Then, the electrophoresis was performed for 30 min at 1.25 V/cm (300 mA). After the electrophoresis, the slides were washed with neutralizing buffer (0.4 M Tris, pH 7.5) and the slides were stained with 2 μg/mL of ethidium bromide for 5 min in dark at 25°C. After the staining, the slides were washed with distilled water, covered with coverslips and imaged by a microscope using the inverted system fluorescence microscope Olympus IX 71S8F-3 (Tokyo, Japan). The excitation filter 520–550 nm and the emission filter of 580 nm were employed (Magnification: 100×) in this experiment. The images were captured by Camera Olympus DP73 and processed by Stream Basic 1.7 Software with the resolution of 4800 × 3600 pixels. From each slide, 10 fields were taken and the nuclei were classified in five categories ranging from 0 (no visible tail) to 4 (most DNA in tail). To quantify the effect of the drugs on DNA damage, an index of damage (ID) was calculated as a sum of the numbers of the nuclei in each category multiplied with 0.1 for category 0 and by 1, 2, 3 and 4 for other categories divided by total number of the nuclei.

$$ID=\frac{0.1\times {\text{N}}_{0}+1\times {\text{N}}_{1}+2\times {\text{N}}_{2}+3\times {\text{N}}_{3}+4\times {\text{N}}_{4}}{{\text{N}}_{0}+{\text{N}}_{1}+{\text{N}}_{2}+{\text{N}}_{3}+{\text{N}}_{4}}$$

### Cultivation of Staphylococcus aureus

*Staphylococcus aureus* (NCTC 8511) was obtained from the Czech Collection of Microorganisms, Faculty of Science, Masaryk University, Brno, Czech Republic. The strains of the bacterium were stored as a spore suspension in 20% (*v/v*) glycerol at −20°C according to the conditions mentioned in Chudobova et al. [36, 37]. Prior to use in this study, the strains were thawed and the glycerol was removed by washing with distilled water. The cultivation medium contained: meat peptone (5 g/L), NaCl (5 g/L), bovine extract (1.5 g/L), yeast extract (1.5 gL) (HIMEDIA, Mumbai, India), and sterilized MilliQ water with 18 MΩ. Before sterilization, the pH of the cultivation medium was adjusted to 7.4 and the media were sterilized at 121°C for 30 min in a sterilizer (Tuttnauer 2450EL, Israel). The prepared cultivation media was inoculated with the bacterial culture into 25 mL Erlenmeyer flasks and subsequently the bacterial cultures were cultivated for 24 h on a shaker at 600 rpm and 37°C. The cultivated bacterial cultures were diluted by the cultivation medium to the optical density (OD_600_) = 0.1 AU and used in the following experiments [38].

### Growth curves

The antimicrobial effect of the tested compounds was analysed by measuring the absorbance using an apparatus Multiskan EX (Thermo Fisher Scientific, Germany) according to Chudobova et al. [39]. Briefly, the concentrations of the platinum compounds (PtNPs and/or cisplatin) were 0, 0.5, 1.0, 1.9, 3.8, 7.5, 15, 30 and 60 μg/mL of Pt. The obtained results were recalculated to relative absorbance value, thus the control growth curve without the addition of the platinum derivatives refers to 100%. The values less than 100% indicated relative cell death.

### Cultivation of human foreskin fibroblast

Human Foreskin Fibroblast (HFF) cell line was obtained from ATCC Cell Lines (Wesel, Germany). HFF cell line was grown as monolayers in DMEM (high glucose) medium with 10% foetal bovine serum, supplemented with penicillin and streptomycin (1 U per mL) at 37°C in 5% CO_2_ atmosphere. Approximately 2 × 10^5^ cells at logarithmic growth phase were treated with free and/or encapsulated CisPt or PtNPs in concentrations 0, 12.5, 25, 50, 100 and 200 μg/mL of Pt for 2h, and harvested using a cell-scraper and resuspended in 200 μL of PBS. After the treatment, the cells were harvested using a cell-scraper and resuspended in 200 μL of PBS. All the experiments were performed in two replicates. HFF were also used for Comet assay.

### Cell growth and proliferation assay of HFF using impedance measurement with xCELLigence system

The xCELLigence system was used according to the instructions of the supplier (Roche Applied Science and ACEA Biosciences). The xCELLigence system consists of four main components: a Real time cell analysis (RTCA) analyser, a RTCA DP station, a RTCA computer with integrated software and a disposable E-plate 16. Firstly, the optimal seeding concentration for the proliferation and RTCA assay of HFF was determined. After seeding the total number of the cells in 200 μL medium in each well of the E plate 16, the attachment, proliferation and spreading of the cells were monitored every 15 min. All the experiments were carried out for 100 h. The obtained results were recalculated to the relative (%) value of the growth. The method of the calculation was same as described for *Staphylococcus aureus*.

### Preparation of erythrocytes for haematological analysis and determination of glutathione

Human blood was obtained from the Department of Physiology, Faculty of Medicine, Masaryk University, Czech Republic. We analysed the samples of healthy volunteers (n = 3), whereas the written consent of blood donors was granted. The research has been approved by the Independent ethics committee (No. 104MS451) at University Hospital, Brno, Czech Republic. The blood samples of three healthy volunteers were collected in the tubes with heparin. The blood samples were centrifuged for 10 min at 2000 rpm. After removing of plasma, the erythrocytes together with platelets were diluted in phosphate buffer pH 7 in a ratio of 1:1. The number of erythrocytes and platelets was determined using haematology analyser BC– 5800 (Mindray, Shenzhen, China). Cisplatin or cisplatin encapsulated in liposomes (LipoCisPt) were added to the erythrocytes mixture with platelets and various concentrations of the Pt derivatives (PtNPs, PtNPs encapsulated in liposomes (LipoPtNPs)) in a ratio of 1:1 to reach the desired concentrations (12.5, 25, 50, 100, 200 μg/mL of Pt and 0.6, 1.3, 2.5, 5 and 10 mg/mL of liposomes) in the resulting mixture. The erythrocytes treated with the platinum compounds were mixed with trifluoroacetic acid (TFA) in a ratio of 2:1 (100 μL of erythrocytes + 50 μL of 15% TFA (*v*/*v*)). Subsequently, the mixture was homogenized using ultrasonic needle for 2 min and vortexed for 10 min. After the centrifugation of the mixture for 20 min at 25 000 rpm, 20 μL of the supernatant was used for chromatographic analysis.

### Estimation of selected haematological parameters of erythrocytes mixture with platelets exposed to platinum derivatives

The haematological analyses of the erythrocytes mixture with platelets exposed to platinum derivatives encapsulated in liposomes or alone were performed using Haematology Analyser BC-5800 (Mindray, Shenzen, China) coupled with flow cytometry. The number of erythrocytes and platelets was determined by impedance method. The flow cytometry together with fluorescence-activated cell sorting was applied to determine the number of leukocytes. The histograms of the erythrocytes mixture with platelets and Pt derivatives encapsulated in liposomes or alone were recorded. The liposomes were visualised in the Baso channel as a red rhombus.

### Determination of reduced and oxidized glutathione

Reduced (GSH) and oxidized (GSSG) glutathione were assayed using a high performance liquid chromatography with electrochemical detection (HPLC-ED) according to Kleckerova et al. [40] and Skladanka et al. [41]. Two solvent delivery pumps (Model 582 ESA Inc., Chelmsford, MA, USA) operating in a range of 0.001–9.999 mL.min^-1^, a column Zorbax eclipse AAA C18 (150 × 4.6; 3.5 μm particle size; Agilent Technologies, Santa Clara, CA, USA) and a CoulArray electrochemical detector (Model 5600A, ESA, USA) with one flow cell were used in this experiment. The cell consists of four working carbon porous electrodes, each one with auxiliary and dry Pd/H_2_ reference electrodes. Both the detector and the reaction coil/column were thermostated. Twenty microliters sample was injected using autosampler (Model 542 HPLC, ESA, USA). The samples were stored in the carousel at 8°C. The column was thermostated at 32°C. The mobile phase was consisted of 80 mM TFA (A) and methanol (B). The compounds of interest were separated by the following linear gradient: 0 → 1 min (3% B, *v*/*v*), 1 → 2 min (10% B, *v*/*v*), 2 → 5 min (30% B, *v*/*v*), 5 → 6 min (98% B, *v*/*v*). The flow rate of the mobile phase was 1 mL.min^-1^ and working electrode potential was set to 900 mV

### Descriptive statistics

Data were processed using MICROSOFT EXCEL^®^ (USA) and STATISTICA.CZ Version 8.0 (Czech Republic). Results are expressed as mean ± standard deviation (S.D.) unless noted otherwise (EXCEL^®^). Statistical significances of the differences were determined using STATISTICA.CZ. Differences with p < 0.05 (labelled with “*” in figures) were considered significant and were determined by using of one way ANOVA test (particularly Scheffe test), which was applied for means comparison.

## Results

### Characterization of PtNPs

The synthesized PtNPs as well as the liposomal formulations were characterized by particle-size and Zeta potential measurement, transmission microscopy imaging, X-ray fluorescence and UV/Vis spectrophotometry and atomic absorption spectrometry. The size of the PtNPs determined by dynamic light scattering was mainly 6.5 nm in diameter with the Zeta potential of -15 kV (Figures A and B in S1 Fig). The X-ray spectrum of the PtNPs was recorded and the two characteristic peaks of Pt Kα (391 keV) and Pt Kβ (457 keV) were observed (Figures A in S2 Fig). The presence of Pt was also confirmed by atomic absorption spectrometry. Subsequently, the stock solution of PtNPs with Pt concentration of 400 μg/mL was prepared and used for further experiments. For these experiments, the stock solution was diluted creating solutions of PtNPs with Pt concentrations ranging from 0 to 200 μg/mL of Pt in PtNPs. The colour of the PtNPs was found to be brown. (Figures B and C in S1 Fig).

### PtNPs and CisPt interactions with DNA fragment from phage λ and Taq DNA polymerase

**Fig 1:**
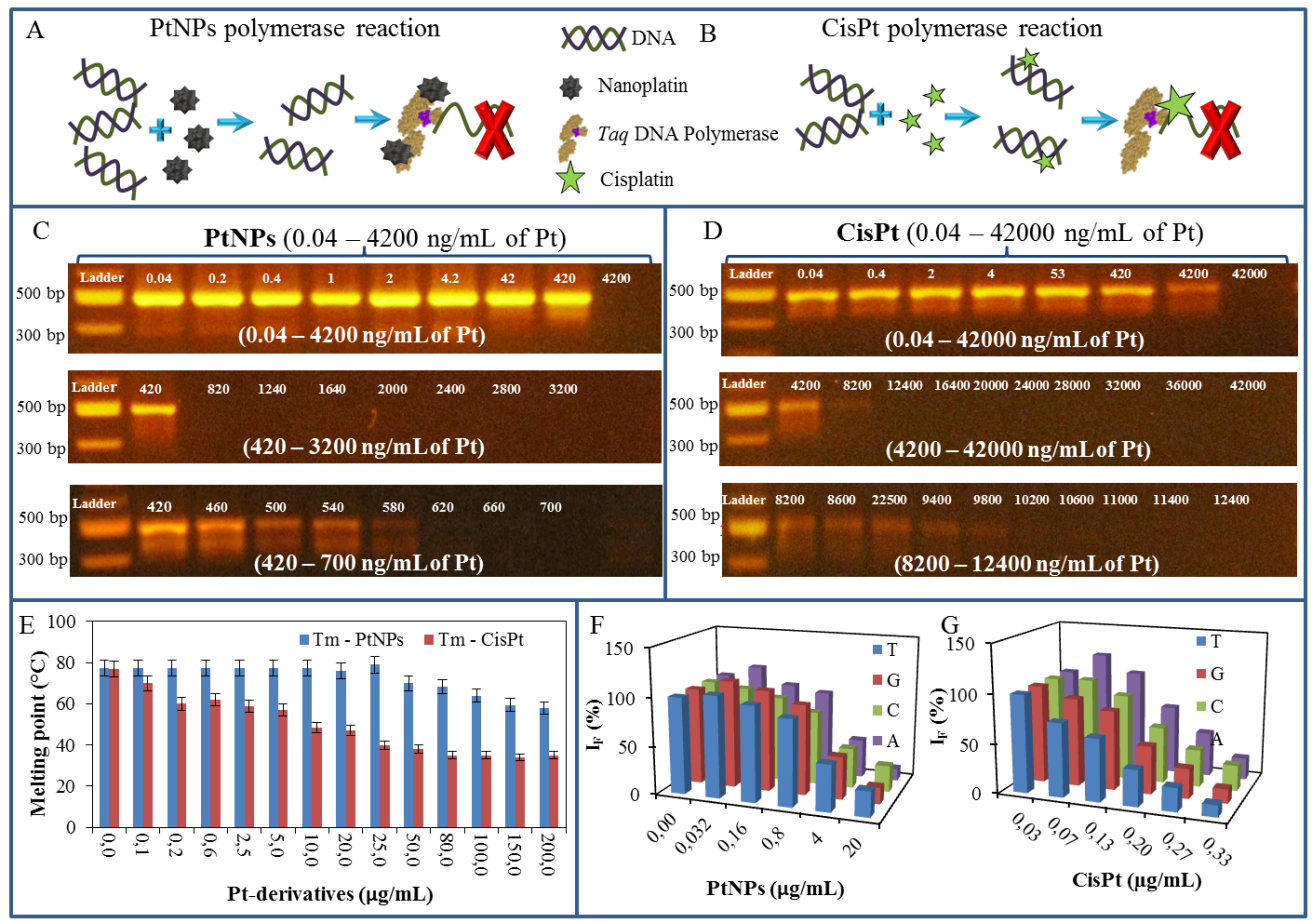
(A) The suggested effects of PtNPs on polymerase chain reaction (PCR) is based on binding of PtNPs to the Taq DNA polymerase, which leads to ceasing of PCR, (B) whereas CisPt primarily intercalates in DNA structure and stops PCR by this way. The gel electrophoregrams of PCR product mixture with particular concentration of (C) PtNPs (0.04–4 200 ng/mL of Pt) and (D) CisPt (0.04–42 000 ng/mL of Pt). (E) DNA denaturation temperature affected by the 0–200 μg/mL of Pt derivatives. Fluorescence of labelled nucleotides of DNA fragment after sequencing, which was influenced by (F) 0–20 μg/mL of PtNPs and (G) 0–0.33 μg/mL of CisPt. For all measurement n = 3.

**Fig 2:**
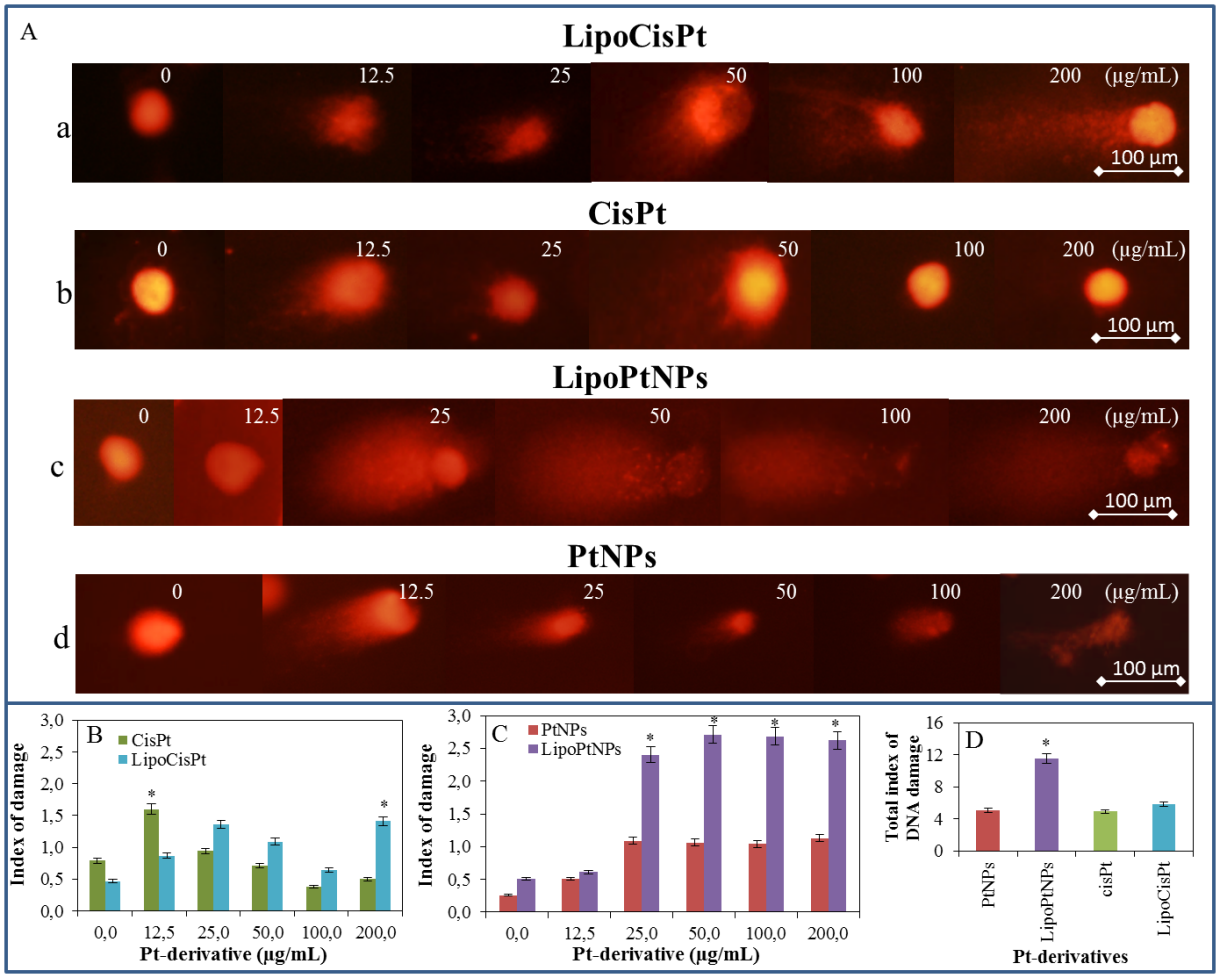
(A) The alkaline comet assay of nuclear DNA from human fibroblasts (HFF) treated with (a) cisplatin (0–200 μg/mL of Pt) encapsulated in liposome LipoCisPt, (b) same concentration of CisPt only, (c) PtNPs encapsulated in liposome LipoPtNPs and (d) free PtNPs. (B) The index of damage of CisPt and LipoCisPt. (C) The index of damage of PtNPs and LipoPtNPs. (D) The nuclear DNA of human fibroblast and summarized their total damage. For all measurement n = 3.

The impact of PtNPs and CisPt on DNA was studied using PCR, DNA melting point (Tm) analysis and Sanger sequencing. It was observed that the function of Taq DNA polymerase was blocked by the PtNPs more effectively than by CisPt. The suggested scheme of the possible mechanism is shown in Figs 1A and 2B.

In the first part of the experiment, it was observed that the PCR was stopped after the addition of at least 0.58 μg/mL PtNPs (Fig 1C). CisPt was studied in the same way and it was found that 9.8 μg/mL CisPt was able to stop the PCR, which is nearly 17-times higher concentration compared to that of the PtNPs (Fig 1D).

In the second part of the experiment, the changes in denaturation temperatures (Tm) were studied. To observe these changes, 5 μg/mL DNA fragments from λ bacteriophage was mixed with the PtNPs or CisPt (0–200 μg/mL). 0.1 μg/mL CisPt decreased the Tm by 7°C compared to the untreated control sample (77°C). To reach the same effect on the Tm, 500 times more PtNPs (50 μg/mL) was needed (Fig 1E). Based on this observation, it was concluded that PtNPs interact more likely with polymerase than with the DNA.

In the third part of the experiment, the DNA fragment of λ bacteriophage (5 μg/mL) was added to the CisPt or PtNPs solutions. These mixtures were subsequently dialysed to remove the particles, which were not interacted or bound to DNA. The modified DNA was used for Sanger sequencing reaction and the DNA was further analysed by the capillary gel electrophoresis with laser-induced fluorescence detection. The fluorescence signals of the probed fragments were compared to the control DNA sample (without the addition of CisPt or PtNPs). Fig 1F shows that the addition of 0.032 μg/mL PtNPs induced the increase in the fluorescence signals of the fragments terminated with the dideoxynucleotides thymine (T), guanine (G) or adenine (A) compared to the control sample. In the case of DNA fragments terminated with the cytosine (C), the decreasing fluorescence was observed even when 0.032 μg/mL PtNPs were added.

After CisPt application, the fluorescence signals of DNA fragments terminated with T and G decreased with the increasing concentration of CisPt. At the DNA fragments terminated with C and A, the fluorescence signal firstly increased (at 0.07 μg/mL CisPt), followed by the decrease in the fluorescence signal with the increasing concentration of CisPt as showed in Fig 1G. The signal decrease by 50% was reached after the application of PtNPs and CisPt in concentrations of 9.6 μg/mL and 0.13 μg/mL, respectively.

From the results of the second and third part of the experiment it is clear that the concentration of the PtNPs should be 500 times (in the case of Tm change) and 74 times (in the case of sequencing) higher than that of CisPt to achieve the same effect in DNA interaction. Conversely, as confirmed by the first experiment, where the PCR mixture contained together with DNA and Taq DNA polymerase, the PCR was stopped by the addition of 580 ng/mL PtNPs, which is 16.9 times lower concentration than that of CisPt. The results of these analyses have unequivocally demonstrated that the PtNPs have a higher affinity to Taq DNA polymerase than to DNA.

### Comet assay

Based on previous experiments, we followed with the cell experiments, where we also tested liposomal forms of both drugs to mimic targeted delivery. Therefore, the extent of DNA damage in the individual somatic cells was studied. To assess the effect of both the PtNPs and CisPt on nuclear DNA of eukaryotic cells the alkaline comet assay was used. The HFF cells in exponential growth phase were exposed to bare and liposome-encapsulated Pt-derivatives in concentrations of 0, 12.5, 25, 50, 100 and 200 μg/mL. Then, the cells were harvested and immobilized into agarose, and lyzed. Later, the nuclei were electrophoresed and stained with ethidium bromide (Fig 2A). The microphotographs show the evident difference between the effect of CisPt (Fig 2Aa and 2Ab) and PtNPs (Fig 2Ac and 2Ad) on the degradation of HFF nuclei. The index of the cell nuclei damage was increased by the encapsulation of CisPt or PtNPs within liposome (Fig 2B and 2C). The liposome-encapsulated PtNPs clearly showed the most intensive degradation effects among all the studied drugs and formulations. As shown in Fig 2C, the platinum nanoparticles (25–200 μg/mL) encapsulated into liposome (LipoPtNPs) exhibited more than two-times higher damage index than that of bare PtNPs. The comparison of the total index of the DNA damage, i.e. mean of indexes of damage presented in Fig 2B and 2C, showed that the PtNPs encapsulated within liposome damaged mostly the cell nuclei (Fig 2D).

### The assessment of PtNPs and CisPt cytotoxicity and the effect of their encapsulation in liposomes

In these experiments we focused on the assessment of the cytotoxicity of the platinum derivatives and also on studying of the effect of their encapsulation in liposomes. Both *S*. *aureus* and HFF were analysed after the application of the PtNPs and CisPt encapsulated in liposomes or alone by the method of growth curves using Multiskan EX (*S*. *aureus*) and xCELLigence (HFF) instruments.

**Fig 3:**
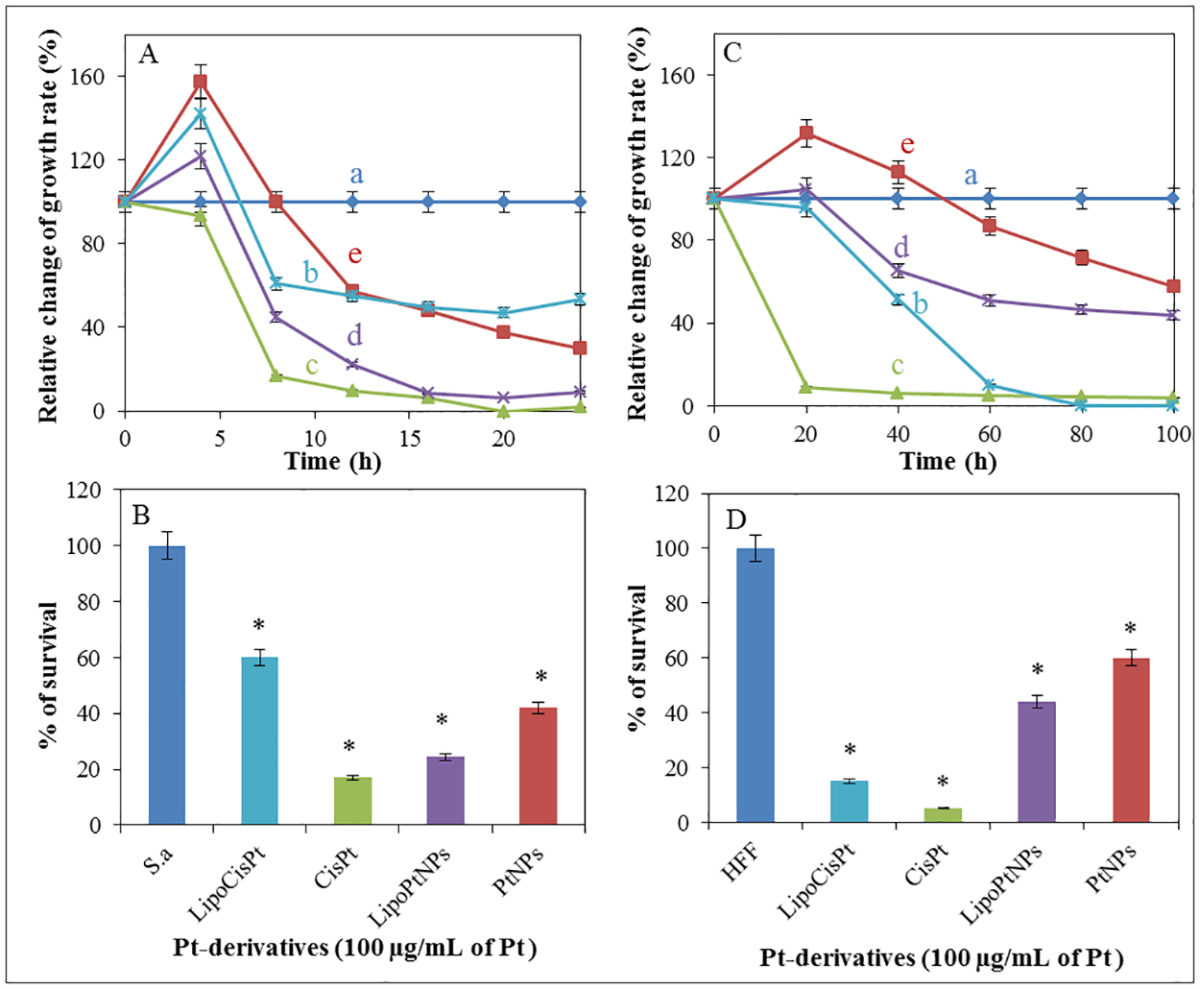
The effect of platinum derivatives on both bacterial and human cells after application of platinum derivatives alone or encapsulated in liposomes. Relative change of growth rate of (A) *S*. *aureus* bacterial culture and (C) HFF after treatment with Pt-derivatives (100 μg/mL of Pt) during 24 h (bacterial culture) and 100 h (HFF) long experiments. (a) Cell culture without any treatment, (b) LipoCisPt, (c) CisPt, (d) LipoPtNPs and (e) PtNPs. Percentage survival of (B) *S*. *aureus* bacterial culture and (D) human foreskin fibroblasts (HFF) after 24 and 100 h of treatment with Pt-derivatives (100 μg/mL of Pt) alone and encapsulated in liposomes. For all measurement n = 3.

Firstly, the growth curves of the bacterial culture were measured after the addition of the Pt-derivatives encapsulated in liposome or alone in concentrations of 100 μg/mL of Pt. The obtained results were recalculated to relative absorbance value, thus, the control growth curve without the addition of the platinum derivatives was considered as 100% (Fig 3Aa). The values lower than 100% indicated relative cell death. The highest cytotoxic effect (100% dead cells) was observed in case of CisPt (Fig 3Ac), whereas a similar effect was achieved after the addition of LipoPtNPs, (Fig 3Ad). After the application of LipoPtNPs, the relative growth rate was found to be decreased for 53% (i.e. 53% dead cells) as compared to that of CisPt. The cytotoxicity of the PtNPs was found to be the lowest among all the drugs during the first 8 h of the treatment (Fig 3Ae), but after 24 h their cytostatic effect was found to be the third most intensive and its value was 20% higher and 30% lower than that of LipoCisPt and CisPt respectively. The results are summarized in Fig 3B, where the cytotoxicity of the Pt-derivatives encapsulated in liposomes or alone are shown.

The growth of HFF was monitored by xCELLigence method in the presence of the platinum derivatives (Fig 3C). The growth of the cells was expressed as percentage of control, thus, the growth curve of the control cells cultivated without the addition of any drug corresponded to 100%. For all tested platinum derivatives, the decrease of the cell growth during the cultivation was observed with exception of PtNPs (Fig 3Ce), where the growth of HFF was found to be stimulated during the first 50 h, but after 50 h the growth inhibition occurred. The highest cytostatic effect (100% decrease) was recorded for CisPt (Fig 3Cc) and LipoCisPt (Fig 3Cb). The PtNPs exhibited markedly lower cytotoxic effect compared to CisPt. After liposome encapsulation, the LipoPtNPs the rate if cell growth inhibition was higher, compared to bare PtNPs. The inhibition activity of LipoPtNPs after 100 h was comparable to the bare PtNPs (Fig 3Cd). The summary of the obtained results is shown in Fig 3D, where the cytotoxicity of the Pt-derivatives encapsulated in liposomes or alone is shown. Based on these results, CisPt had the highest cytostatic and cytotoxic effect for both *S*. *aureus* and HFF. The encapsulation of cisplatin in liposomes decreased both cytostatic and cytotoxic effects of cisplatin for the bacterial cells as well as for HFF. The PtNPs were found to be more effective in the bacterial cells than in HFF. The encapsulation in liposomes increased both the cytostatic and cytotoxic effects of the PtNPs, where one may suggest one of the following four mechanisms of liposome-cell interaction by which liposomes deliver their content in cells can occur: i) adsorption followed by extracellular release of liposome content; ii) endocytosis clathrin-(in) dependent; iii) lipid exchange by transfer of lipophilic compounds from the liposomal bilayer to the cell membrane and iv) fusion with the intracellular membrane.

### The influence of Pt derivatives encapsulated in liposomes and alone on erythrocytes mixture with platelets (flow cytometry and GSH/GSSG ratio)

The commonly used cytostatics such as CisPt as well as their new alternatives such as PtNPs are often administered intravenously and therefore they may interact with erythrocytes and platelets.

**Fig 4:**
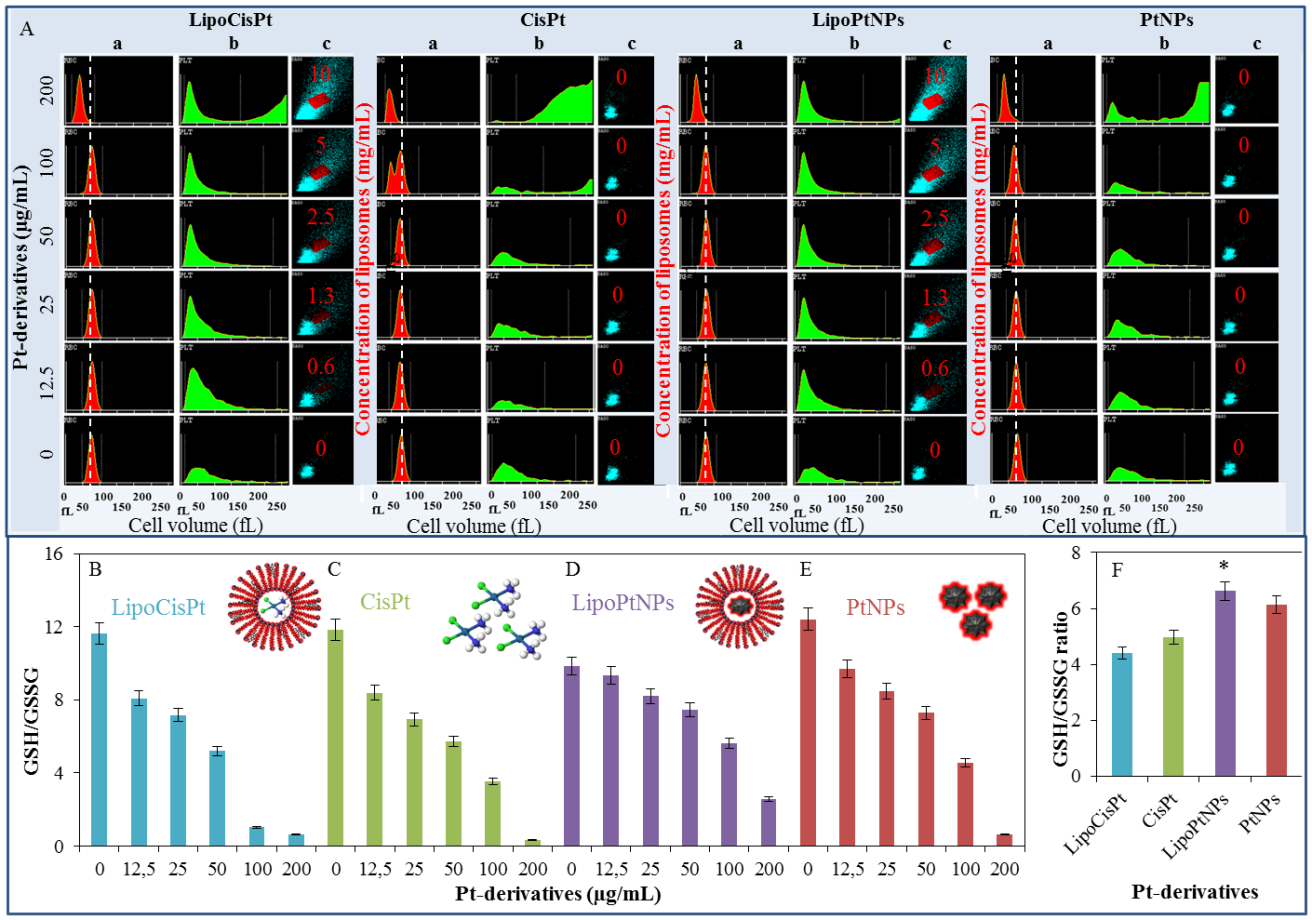
Selected haematological parameters of erythrocytes mixture with platelets and oxidative stress (GSH/GSSG) in the mixture after application of Pt derivatives encapsulated in liposomes or alone in concentrations 0, 12.5, 25, 50, 100 and 200 μg/mL of Pt and 0, 0.6, 1.3, 2.5, 5 and 10 mg/mL of liposomes. (A) Histograms of (a) erythrocytes and (b) platelets, and (c) visualisations of liposomes in the Baso channel after application of LipoCisPt, CisPt, LipoPtNPs and PtNPs. GSH/GSSG ratio of erythrocytes mixture with platelets in the same concentrations as in A—(B) LipoCisPt, (C) CisPt, (D) LipoPtNPs, and (E) PtNPs. (F) Mean of GSH/GSSG ratio. For all measurement n = 3, significant difference is indicated by *p<0.05.

The influence of the platinum derivatives encapsulated in liposomes or alone on human erythrocytes mixed with platelets was studied by flow cytometry using haematology analyser and the oxidative stress was determined by GSH/GSSG ratio. The mixtures of the erythrocytes and platelets were exposed to LipoCisPt, CisPt, LipoPtNPs and PtNPs in concentrations of 0, 12.5, 25, 50, 100 and 200 μg/mL of Pt for 16 h. The concentrations of the liposomes in LipoPtNPs and LipoCisPt were as follows: 0, 0.6, 1.3, 2.5, 5, and 10 mg/mL. The histograms of the mixture and the Pt derivatives encapsulated in liposomes or alone were recorded by a haematology analyser. The liposomes were visualised in the Baso channel as a red rhombus (Fig 4A). No significant interaction was found for any of the Pt derivatives and the erythrocyte-platelet mixture.

The PtNPs encapsulated in liposomes were prepared and subsequently diluted decreasing the concentration of both components concurrently. The effect of this treatment is shown in Fig 4Ac.

The GSH and GSSG concentrations were determined by HPLC-ED (Fig 4B–4E). After treatment by all the Pt derivatives, a decrease in the GSH/GSSG ratio was observed that indicates the increasing amount of the oxidative stress in the erythrocytes. From Fig 4F, it can be clearly seen that LipoCisPt and CisPt caused higher oxidative stress than LipoPtNPs and PtNPs. By the statistical analysis using Lord´s T test, it was proven that compounds containing CisPt statistically differ from compounds containing PtNPs. However, the statistically significant difference was detected also between CisPt and PtNPs as well as between LipoCispt and LipoPtNPs. At the confidence level of 0.05.

## Discussion

**Fig 5:**
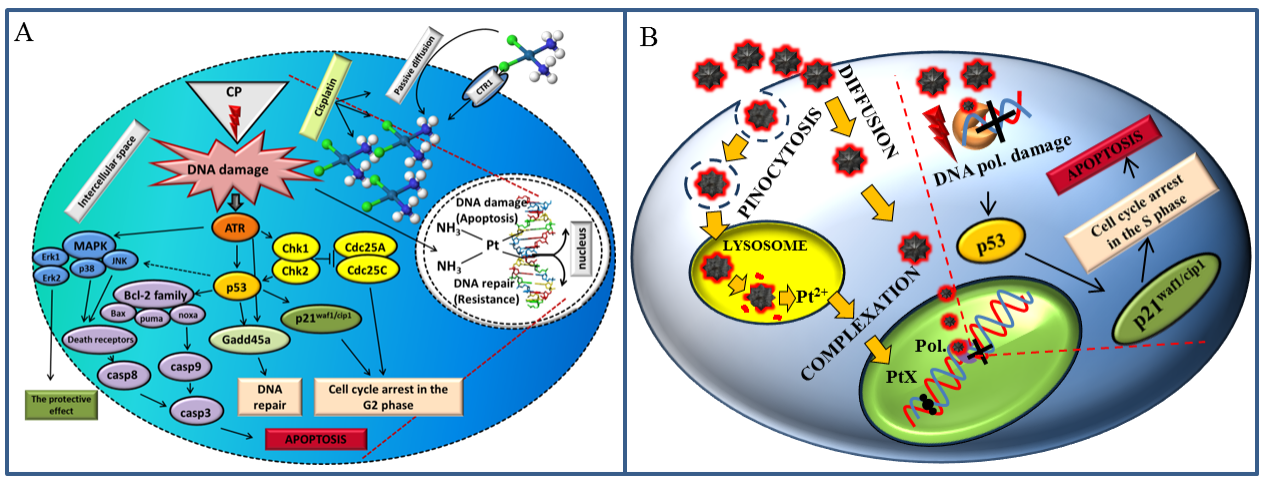
(A) Effect of CisPt on the somatic cell. (B) Proposed effect of PtNPs on the somatic cell. Figure prepared according to [54].

In the present experiment, the effects of PtNPs, or CisPt alone and encapsulated in liposomes (LipoPtNPs or LipoCisPt) on bacterial cultures (*Staphylococcus aureus)*, HFF, erythrocytes and DNA (PCR product of *Xis* gene of λ phage) were studied. The platinum complexes are the active substance used as effective cytostatics [14]. Hundreds of Pt(II) and Pt(IV) complexes have been synthesized since the discovery the antibacterial effects of platinum in 1965 [42–44]. The effect of the platinum complexes (cisplatin, carboplatin and oxaliplatin) is most probably based on their covalent binding with DNA bases [45, 46] forming intra- and interstrand crosslinks, DNA—protein crosslinks, and monoadducts with DNA [47]. DNA secondary structures can block transcription and replication with subsequent apoptosis [48]. Pt drugs enter cells mainly via passive diffusion, although some evidence of its active transport by Ctr1 system involved in the maintaining of copper homeostasis was reported (Fig 5A) [49–52]. Another possibility is the intake of the nanoparticles *via* endocytosis [53]. Cisplatin-induced DNA damage activates ATR kinase, which triggers the effector molecules. One of the ATR kinase targets is a tumour suppressor protein p53, which is phosphorylated by the kinase on serine 15. This phosphorylation causes the decrease of p53 affinity to its negative regulator Mdm-2, which leads to an increased p53 concentration. The protein p53 then initiates the transcription of p21 gene, which inhibits the cyclin-dependent kinases Cdk2 and Cdk4 and consequent arrests the cell cycle. p53 also induces the expression of pro-apoptotic members of Bcl-2 family such as Bax, Puma and Noxa in response of the activation of mitochondrial apoptotic pathway.

On the mitochondrial surface, these pro-apoptotic proteins meet the anti-apoptotic Bcl-2 family members. The main determinant of cisplatin-induced DNA damage is the ratio of pro-apoptotic and anti-apoptotic proteins. The majority of the pro-apoptotic signals lead to the release of cytochrome c from mitochondria followed by the activation of cysteine caspases selectively degrading the target proteins. Initiator caspases (Casp-8, 9 and 10) activate the effector caspases (Casp-3, 6 and 7). Casp-8 and Casp-9 are activated by outer pathway through the death receptors and inner mitochondrial pathway respectively. Both Casp-8 and 9 then activate Casp-3, which is responsible for intrinsic apoptotic process [53, 55, 56].

Among thousands of published papers aimed at the mentioned platinum-based cytostatics, only few can be found with the physiological action of the platinum nanoparticles [57, 58]. Some papers inform about the antioxidant properties of the PtNPs. Kajita et al. reported that PtNPs eliminated the anion radicals and hydrogen peroxide by a mechanism similar to superoxide-dismutase and catalase action [18]. Kim et al. [59] also confirmed the antioxidant function of PtNP,s which were shown to reduce the oxidative stress in murine osteoclasts. Onizawa et al. [60], studied the antioxidant activity of PtNPs in murine lungs after exposition to cigarette smoke. In a separate experiment, synthetic PtNPs at ppb levels were shown to extend the nematode lifespan and scavenge ROS which was induced by paraquat treatment in the nematode [61]. The anti-inflammatory effects of platinum nanoparticles on the lipopolysaccharide-induced inflammatory response in RAW 264.7 macrophages were determined by Rehman et al. [62].

Oh et al. reported the synthesis of the PtNPs of a diameter of 4.8 nm [29], which were characterized by X-ray and UV/Vis spectrophotometry [63]. In this work, the PtNPs were shown (using different techniques like PCR, DNA sequencing and denaturation) to inhibit the *Taq* DNA polymerase and affect the secondary structure of DNA in higher concentrations. Different papers on the interaction of heavy metals (Cu, Pb, and Cd) with DNA polymerase and thus inhibition of PCR were previously published [64, 65]. Our findings are consistent with earlier reports emphasizing intracellular release of Pt^2+^ ions from PtNPs, which could block cell division by binding with DNA [66]. The intracellular interactions of the PtNPs with bacterial DNA (*Salmonella Enteritidis*) were also previously reported [27] and it was shown using DNA-binding fluorochrome that one hour treatment of *Salmonella enteritidis* with PtNPs removed part of the DNA from the bacterial cell [27]. Shukla et al. showed that the nanoparticles tend to aggregate in the liposomes [67]. The aggregation of the PtNPs in liposomes may be responsible for the observed PtNPs cytotoxicity. This phenomenon can be explained by the oxidation of Pt by hydrogen peroxide according to the equation: Pt + H_2_O_2_ + 2H^+ →^ Pt^2+^ + 2H_2_O [67]. The different mechanisms of CisPt and PtNPs action were found by single cell gel electrophoresis (comet assay) in this study. This method allows us to detect DNA damage on the single cell level [68]. Different modes of HFF nuclei degradation were observed after the treatment with CisPt or PtNPs, where the PtNPs were found to cause a higher degree of DNA degradation compared to CisPt. This finding may be caused by the fact that the alkaline comet assay is more sensitive to dsDNA breakage than to ssDNA breakage. In case of CisPt, ssDNA breakage [69], and covalent binding to DNA and interstrand crosslink [45] were observed as a result of a direct interaction with the DNA. The dsDNA breakages were caused rather by the PtNPs-induced Pt^2+^ ions formed during the incubation of the PtNPs with DNA [23].

The interactions of the Pt-derivatives with DNA might cause the arrest of cell cycle or have mutagenic effect [70]. The results suggest that p53 was activated in PtNPs treated cells due to the genotoxic stress with subsequent activation of p21 leading to a proliferating cell nuclear antigen-mediated growth arrest and apoptosis (Fig 5B) [54]. In this study, an attention was also paid to the antibacterial effects of the nanomaterials [71]. The PtNPs seemed to be a significant antibacterial agent [26, 72]. According to Chwalibog et al. PtNPs cause the degradation of cytoplasmic membrane and bacterial cell wall [26]. Our results indicated that the encapsulation of the nanoparticles in liposomes may lead to a more effective delivery of the nanoparticles as well as the disruption of the cellular membranes and thus increase their cytotoxicity and antibacterial action [73, 74].

In this work, the inhibition of bacterial and HFF growth by PtNPs and CisPt alone and encapsulated in liposome was compared by the method of growth curves and it was found that the inhibition effect of LipoPtNPs on *S*. *aureus* cells was higher compared to bare PtNPs. The inhibition of both keratinocytes and bacterial growth by PtNPs was also reported previously by Konieczny et al. [53], where the authors found an increased DNA damage and caspase 9 activity in human keratinocytes and a size-dependent inhibition effect of PtNPs on *S*. *aureus* and *E*. *coli* using a colony-reduction assay. No differences between PtNPs and CisPt action on human erythrocytes was found by haematological analyser. Both the bare and encapsulated PtNPs were found to cause significantly lower oxidative stress in the erythrocytes compared to the bare and encapsulated CisPt (as measured by GSH/GSSG ratio).

## Conclusions

In this paper, we showed that PtNPs primarily inhibit the activity of *Taq* DNA polymerase and damage to the DNA structure. We tend to believe that this effect together with the transition of PtNPs to Pt^2+^ causes mutagenicity and increases DNA damage compared to CisPt. The cytotoxic effect of the PtNPs may be increased by their encapsulation in liposome. The results suggest that the activation of p53 in PtNPs treated cells was caused by the genotoxic stress with subsequent activation of p21 leading to a proliferating cell nuclear antigen-mediated growth arrest in S phase and following apoptosis [54]. It is conceivable that the PtNPs with the effective antitumor activity may provide an alternative treatment for cancer.

## Supporting information

S1 Text Supporting materials and methods. The following experimental details are shown there Particle size and zeta-potential analysis (PtNPs and Liposomes), Particle size assessment, Zeta potential assessment, Transmission electron microscopy (PtNPs and Liposomes), X-ray fluorescence analysis (XRF), Atomic absorption spectrometry and UV/vis spectrophotometry.
(DOCX)

S1 Fig Characterization of the particles. A) Dynamic light scattering analysed of PtNPs in PBS, pH 7.4, with corresponding ζ potential inserted. B) TEM micrograph (length of scale bar is 50 nm) of and PtNPs. C) Dynamic light scattering analysed of Liposomes in PBS, pH 7.4, with corresponding ζ potential inserted. D) TEM micrograph (length of scale bar is 200 nm) of Liposomes.
(DOCX)

S2 Fig Spectral characterization of PtNPs. A) X-ray fluorescence (XRF) spectrum of 200 μg/mL the platinum nanoparticles (PtNPs). B) The absorption spectrum of polyvinylpyrrolidone (PVP); in inset: chemical formula of PVP basic unit—pyrrolidone. C) The absorption spectra of PtNPs (a = 200, b = 100, c = 50, d = 25, e = 12.5, f = 6.3, g = 3.2 and h = 0 μg/mL of Pt). D) PtNPs visualized in ambient light measured in the same concentrations as in S1 Fig.
(DOCX)
