# Supplementary material for: Platinum nanoparticles induce damage to DNA and inhibit DNA replication
Source: PLoS One. 2017 Jul 12;12(7):e0180798. doi: 10.1371/journal.pone.0180798 (PMC5507526; doi:10.1371/journal.pone.0180798)
Supplement: S1 Text — The following experimental details are shown there Particle size and zeta-potential analysis (PtNPs and Liposomes), Particle size assessment, Zeta potential assessment, Transmission electron microscopy (PtNPs and Liposomes), X-ray fluorescence analysis (XRF), Atomic absorption spectrometry and UV/vis spectrophotometry. (DOCX) [file pone.0180798.s001.docx]

**Platinum nanoparticles induce damage to DNA and inhibit DNA replication**

Lukas Nejdl^1,2^, Jiri Kudr^1,2^, Amitava Moulick^1,2^, Dagmar Hegerova^1,2^, Branislav Ruttkay-Nedecky^1,2^, Jaromir Gumulec^2,3^, Kristyna Cihalova^1,2^, Kristyna Smerkova^1,2^, Simona Dostalova^1,2^, Sona Krizkova^1,2^, Marie Novotna^1,2^, Pavel Kopel^1,2^, Vojtech Adam^1,2*^

*^1^Department of Chemistry and Biochemistry,* *Mendel University in Brno,* *Zemedelska 1, CZ-613 00 Brno, Czech Republic*

*^2^Central European Institute of Technology, Brno University of Technology, Purkynova 1, CZ-612 00 Brno, Czech Republic*

*^3^Department of Pathological Physiology, Faculty of Medicine,* *Masaryk University, Kamenice 5, CZ-625 00 Brno, Czech Republic*

***Corresponding author**

E-mail: [vojtech.adam@mendelu.cz](mailto:vojtech.adam@mendelu.cz)

**S1Text. Supporting Materials and Methods**

**Particle size and zeta-potential analysis (PtNPs and Liposomes)**

Zetasizer MALVERN, Malvern Instruments Ltd. Worcestershire WR14 1XZ, United Kingdom was used.

**Particle size assessment**

The particle size measurements were performed considering a refraction index of the dispersive phase of 2.188 and 1.333 for the dispersive environment. The absorption coefficient in both cases was 10-3. The measuring temperature was set at a constant value of 25 °C, while the viscosity was 0.8872 cP. For each measurement, disposable cuvettes type ZEN 0040, were used, containing 40 µL of sample. The equilibration time was 120 s, at a measurement angle of 173° backscatter. All measurements were triplicate (p < 0.05) and the data was expressed as the average value.

**Zeta potential assessment**

Measuring parameters such as, temperature and viscosity were the same as in particle size measurements calculations considered the diminishing of particles concentration based Smoluchowsky model, with a F(κa) of 1.50 and an equilibrating time of 120 s. For the measurements, a disposable cell DTS1070 was employed. In each case, the measurement duration depended on the number of runs, which varied between 20 and 40. The measurements were carried out in triplicates and were performed under the automatic setting of attenuation and voltage selection.

**Transmission electron microscopy (PtNPs and Liposomes)**

For documentation of structure, MIRA2 LMU (Tescan, Brno, Czech Republic) fitted with In-Beam SE detector. An accelerating voltage of 5 kV gave satisfactory results regarding maximum throughput. TEM analyses were performed using the sample (~4 μL) deposited onto 400-mesch copper grids coated with a continuous carbon layer. Dried grids were imaged by Tecnai F20 TEM (FEI, Eindhoven, Netherlands) at 120 kV.

**X-ray fluorescence analysis (XRF)**

The PtNPs were analysed using X-ray fluorescence analysis using Spectro Xepos (Spectro Analytical Instruments, Kleve, Germany). The measurement time was 300 s. For excitation a Mo secondary target was used. The sample was measured through the PE bottle sidewall 20 mm above the bottom. The obtained data were analysed using Spectro Xepos software and TurboQuant method.

**UV/vis spectrophotometry**

The spectra of the PtNPs or CisPt (0 - 400 µg/mL) and 1 mM PVP were recorded within the wavelength range from 200 to 800 nm using quartz cuvettes (1 cm, Hellma, Essex, UK) and a spectrophotometer SPECORD 210 (Analytik Jena, Jena, Germany) at 25 °C maintained by Julabo (Labortechnik, Wasserburg, Germany). The denaturation of the DNA complex with PtNPs or CisPt in the concentration range 0 - 1 mM was monitored using a spectrophotometer SPECORD S600 with a diode detector (Analytik Jena, Jena, Germany). The sample was incubated for 3 min within the temperature range from 25 to 99 °C and the absorbance was measured within the wavelength range from 200 to 800 nm. The changes in absorbance spectra of the complex were recorded and evaluated by WinASPECT (version 2.2.7.0).

**Atomic absorption spectrometry**

Platinum was determined by 280Z Agilent Technologies atomic absorption spectrometer (Agilent, Santa Clara, USA) with electrothermal atomization. A platinum hollow cathode lamp (Agilent) was used as a radiation source. The spectrometer was operated at 265.9 nm resonance line with spectral bandwidth of 0.2 nm and the lamp current of 10 mA. After the turning on the heating time–temperature program, a sample volume of 20 µL was injected into the tube. The pyrolysis temperature of 1000 °C for 8 s and the atomization temperature of 2700 °C for 3 s were applied. The argon inert gas flew at a speed of 300 mL/min. Zeeman background correction was used with field strength of 0.8 T. The absorption signal was evaluated in peak height mode with 7-point smoothing. Each of the samples was measured in triplicates.
