# Supplementary material for: Platinum nanoparticles induce damage to DNA and inhibit DNA replication
Source: PLoS One. 2017 Jul 12;12(7):e0180798. doi: 10.1371/journal.pone.0180798 (PMC5507526; doi:10.1371/journal.pone.0180798)
Supplement: S1 Fig — A) Dynamic light scattering analysed of PtNPs in PBS, pH 7.4, with corresponding ζ potential inserted. B) TEM micrograph (length of scale bar is 50 nm) of and PtNPs. C) Dynamic light scattering analysed of Liposomes in PBS, pH 7.4, with corresponding ζ potential inserted. D) TEM micrograph (length of scale bar is 200 nm) of Liposomes. (DOCX) [file pone.0180798.s002.docx]

**Platinum nanoparticles induce damage to DNA and inhibit DNA replication**

Lukas Nejdl^1,2^, Jiri Kudr^1,2^, Amitava Moulick^1,2^, Dagmar Hegerova^1,2^, Branislav Ruttkay-Nedecky^1,2^, Jaromir Gumulec^2,3^, Kristyna Cihalova^1,2^, Kristyna Smerkova^1,2^, Simona Dostalova^1,2^, Sona Krizkova^1,2^, Marie Novotna^1,2^, Pavel Kopel^1,2^, Vojtech Adam^1,2*^

*^1^Department of Chemistry and Biochemistry,* *Mendel University in Brno,* *Zemedelska 1, CZ-613 00 Brno, Czech Republic*

*^2^Central European Institute of Technology, Brno University of Technology, Purkynova 1, CZ-612 00 Brno, Czech Republic*

*^3^Department of Pathological Physiology, Faculty of Medicine,* *Masaryk University, Kamenice 5, CZ-625 00 Brno, Czech Republic*

***Corresponding author**

E-mail: [vojtech.adam@mendelu.cz](mailto:vojtech.adam@mendelu.cz)

triplicates.

**S1 Fig. Characterization of the particles.**


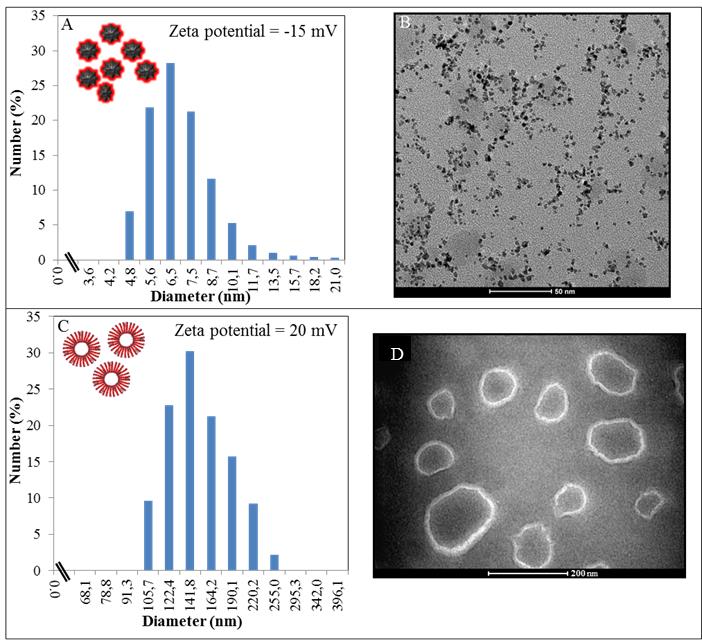


**S1 Fig.** **Characterization of the particles.** A) Dynamic light scattering analysed of PtNPs in PBS, pH 7.4, with corresponding ζ potential inserted. B) TEM micrograph (length of scale bar is 50 nm) of PtNPs. C) Dynamic light scattering analysed of Liposomes in PBS, pH 7.4, with corresponding ζ potential inserted. D) TEM micrograph (length of scale bar is 200 nm) of Liposomes.
