# Supplementary material for: Platinum nanoparticles induce damage to DNA and inhibit DNA replication
Source: PLoS One. 2017 Jul 12;12(7):e0180798. doi: 10.1371/journal.pone.0180798 (PMC5507526; doi:10.1371/journal.pone.0180798)
Supplement: S2 Fig — A) X-ray fluorescence (XRF) spectrum of 200 μg/mL the platinum nanoparticles (PtNPs). B) The absorption spectrum of polyvinylpyrrolidone (PVP); in inset: chemical formula of PVP basic unit—pyrrolidone. C) The absorption spectra of PtNPs (a = 200, b = 100, c = 50, d = 25, e = 12.5, f = 6.3, g = 3.2 and h = 0 μg/mL of Pt). D) PtNPs visualized in ambient light measured in the same concentrations as in S1 Fig. (DOCX) [file pone.0180798.s003.docx]

**Platinum nanoparticles induce damage to DNA and inhibit DNA replication**

Lukas Nejdl^1,2^, Jiri Kudr^1,2^, Amitava Moulick^1,2^, Dagmar Hegerova^1,2^, Branislav Ruttkay-Nedecky^1,2^, Jaromir Gumulec^2,3^, Kristyna Cihalova^1,2^, Kristyna Smerkova^1,2^, Simona Dostalova^1,2^, Sona Krizkova^1,2^, Marie Novotna^1,2^, Pavel Kopel^1,2^, Vojtech Adam^1,2*^

*^1^Department of Chemistry and Biochemistry,* *Mendel University in Brno,* *Zemedelska 1, CZ-613 00 Brno, Czech Republic*

*^2^Central European Institute of Technology, Brno University of Technology, Purkynova 1, CZ-612 00 Brno, Czech Republic*

*^3^Department of Pathological Physiology, Faculty of Medicine,* *Masaryk University, Kamenice 5, CZ-625 00 Brno, Czech Republic*

***Corresponding author**

E-mail: [vojtech.adam@mendelu.cz](mailto:vojtech.adam@mendelu.cz)

**S2 Fig. Spectral characterization of PtNPs**

**
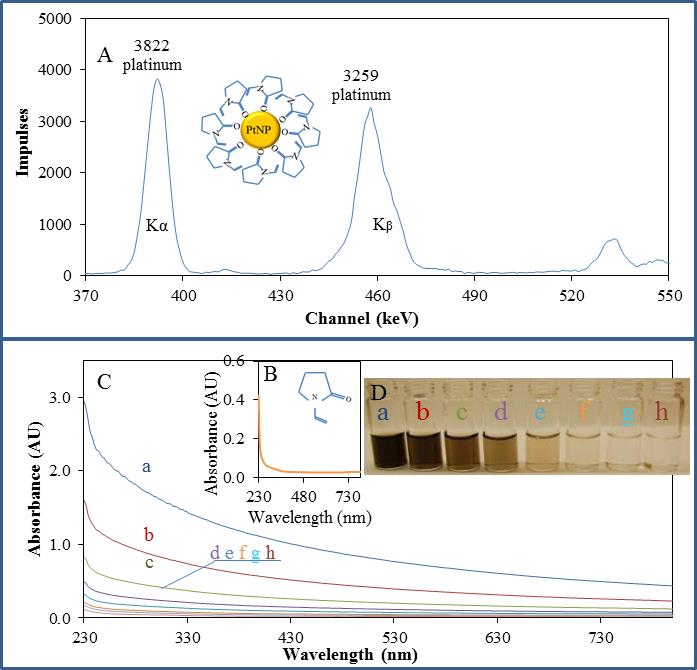
**

**S2 Fig. Spectral characterization of PtNPs.** A) X-ray fluorescence (XRF) spectrum of 200 µg/mL the platinum nanoparticles (PtNPs). B) The absorption spectrum of polyvinylpyrrolidone (PVP); in inset: chemical formula of PVP basic unit - pyrrolidone. C) The absorption spectra of PtNPs (a = 200, b = 100, c = 50, d = 25, e = 12.5, f = 6.3, g = 3.2 and h = 0 µg/mL of Pt). D) PtNPs visualized in ambient light measured in the same concentrations in S2 Fig
